# Melting Behaviors of Bio-Based Poly(propylene 2,5-furan dicarboxylate)-b-poly(ethylene glycol) Co Polymers Related to Their Crystal Morphology

**DOI:** 10.3390/polym16010097

**Published:** 2023-12-28

**Authors:** Ouyang Shi, Peng Li, Chao Yang, Haitian Jiang, Liyue Qin, Wentao Liu, Xiaolin Li, Zhenming Chen

**Affiliations:** 1 School of Materials Science and Engineering, Guilin University of Technology, Guilin 541004, China; shiouyang2022@163.com (O.S.); yangchao_chem@163.com (C.Y.); lwt521021@163.com (W.L.); 2 Guangxi Key Laboratory of Comprehensive Utilization of Calcium Carbonate Resources, College of Materials and Environmental Engineering, Hezhou University, Hezhou 542899, China; 17377109132@163.com (H.J.); 15277812331@163.com (L.Q.); 3 Guangxi HuaLong Resin Co., Ltd., Hezhou 542899, China; ixl@hlupr.com

**Keywords:** crystallization and melt, random block copolymer, poly(dimethyl 2,5-furandicarboxylate), crystalline morphology, cyclic ring-banded spherical crystal, biodegradable polymer

## Abstract

In this experiment, a series of poly(propylene 2,5-furan dicarboxylate)-b-poly(ethylene glycol) (PPFEG) copolymers with different ratios were synthesized using melt polycondensation of dimethylfuran-2,5-dicarboxylate (DMFD), 1,3-propanediol (PDO) and poly(ethylene glycol) (PEG). The effect of PEG content on the crystallization behavior of the poly(propylene 2,5-furan dicarboxylate) (PPF) copolymers was investigated. For PPF, the nucleation density of the *β*-crystals was higher than that of *α*-crystals. As *T*_c_ increases, the *β* crystals are suppressed more, but at *T*_c_ = 140 °C, the bulk of PPF has already been converted to *α* crystals, which crystallize faster at higher nucleation densities, resulting in a difference in polymer properties. For this case, we chose to add a soft segment material, PEG, which led to an early multi–melt crystallization behavior of the PPF. The addition of PEG led to a decrease in the crystallization temperature of PPF, as well as a decrease in the cold crystallization peak of PPF. From the crystalline morphology, it can be seen that the addition of PEG caused the transformation of the PPF crystalline form to occur earlier. From the crystalline morphology of PPF at 155 °C, it can be observed that the ring-banded spherical crystals of the PPF appear slowly with increasing time. With the addition of PEG, spherical crystals of the ring band appeared earlier, and even appeared first, and then disappeared slowly.

## 1. Introduction

Polyesters (such as PET) with terephthalic acid (PTA) as the main raw material have many excellent properties. This is because of the presence of rigid benzene rings in the main chain of terephthalic acid (PTA) [1,2,3]; thus, the mechanical properties, gas barrier, and thermal properties are good. The chemical structure and properties of 2,5-furan dicarboxylic acid (FDCA) and terephthalic acid (PTA) are similar [4,5,6]. Therefore, furan-based polyesters are attracting widespread attention as ideal materials to replace PET-like materials in the future and are very important in the field of biodegradable materials [7,8,9]. Additionally, due to the presence of the polar furan ring on the main chain, its gas barrier properties are particularly outstanding; most importantly, as the monomer FDCA for the synthesis of polyesters can be obtained from biomass–derived materials, the furan dicarboxylic acid group of polyesters is, therefore, an extremely promising bio–based alternative material to aromatic petroleum–based polyesters [10]. However, poly(1,3-propylene 2,5-furan dicarboxylate) (PPF) is a full bio–based polyester material of great interest due to its structural properties similar to those of propylene terephthalate (PTT), high modulus, high glass transition temperature, and high gas barrier; however, PPF also suffers from the inherent defects of furanyl dicarboxylate polyester materials such as brittleness and low crystallization rate [11,12]. The usual method used to modify these shortcomings is copolymerization. Although a great deal of work has been reported on PPF, little research has been carried out on its crystallization behavior and mechanisms. Notably, crystallization phenomena are very important for polymeric materials, as they determine the final degree of crystallinity and morphology of the final product after processing. In other words, they determine several parameters of the final polymeric material, which are crucial for their application. Therefore, it is of interest to investigate the exploration of crystallization phenomena [13,14,15].

Zhang et al. [16] found that the crystallization of PES fragments in PES-b-PTEGT was significantly enhanced by interface–assisted crystallization under different crystallization conditions, which led to a significant improvement in the mechanical properties of PES-b-PTEGT. Therefore, crystal morphology is very important for polymer materials because it determines the properties of the final product after processing. In other words, they determine several of the most important parameters of the final polymer material. The crystal structure plays a decisive role in determining the properties of the material. Materials with different properties can be obtained using different crystal shapes.

For the crystallization behavior of pure PPF, in 2020, Maria Cristina Righetti et al. [17] conducted an in-depth analysis of the crystal structure of poly(propylene 2,5-furan dicarboxylic acid ester) (PPF) after removing the catalyst to the maximum extent. The study revealed that purified PPF presents two different crystalline phases after crystallization from the melt. Crystallization at temperatures below 120 °C led to the formation of a single–crystal type (*α*-type). At 160 °C, imperfect *β*’ crystals can be transformed into more perfect *β*. Two different crystal types (*α* and *β*) can coexist at different crystallization temperatures. For the crystallization behavior of PPF copolyesters, in 2020, Xue Weiwei et al. [18] synthesized a random copolymer of poly(propylene succinate propylene fumarate) (PPSF) with 1,3-propanediol as the diol source. PPSF exhibits a novel cocrystallization behavior that has rarely been discovered, which would combine the advantages of both isomorphism and isodimorphism. In addition, the alteration of the PPS-like to PPF-like crystal structure of PPSF when the chain composition is changed has been proven to originate from the shift in the dominant intersegment interaction from van der Waals forces to strong hydrogen–bonding interactions. This work enriches our understanding of the co–crystallization manner of random copolymers. For the crystallization behavior of PPF composites, in 2021, Wang et al. [19] prepared bio–based PPF/CNTs nanocomposites by a solution and coagulation method at low CNT loadings of 0.5 to 2 wt%. When the amount of carbon nanotubes was 2 wt%, PPF exhibited obvious exothermic crystallization at 123.4 °C, and the crystallization enthalpy was relatively high, which was 29.7 J/g. CNTs not only promote the crystallization behavior of the melt at a constant cooling rate but also improve the isothermal crystallization rate at the same crystallization temperature as the nucleating agent. From a sustainability perspective, incorporating a bio–based material into the PPF matrix is of great significance [20].

In this paper, a series of PPFEG copolymers with different ratios were synthesized via melt polycondensation of dimethylfuran-2,5-dicarboxylate (DMFD), 1,3-propanediol (PDO), and polyethylene glycol (PEG). The effect of the PEG ratio on the crystallization behavior and multiple melting behaviors of the PPFs was analysed. The melting behavior of PPF and PPFEG copolymers was investigated to analyse how the crystal structure and crystalline morphology of the PPF and PPFEG copolymers reorganized during heating and to determine the relative stability of the various crystalline forms. In addition, the crystal morphology was observed by polarized light microscopy (POM), and the effect of the soft–segment material PEG on the crystalline behavior of the PPFs was analysed by employing crystalline morphology. The crystallization rate of PPF is relatively slow, and the addition of the soft section material PEG makes it possible to study whether the crystallization phenomenon of PPF can be brought forward and to study the effect of different contents of PEG on the crystallization performance of PPF to enrich the content of the PPF field.

## 2. Materials and Methods

### 2.1. Materials

Dimethylfuran-2,5-dicarboxylate (DMFD 99.5%) was purchased from ChemTarget Co., Ltd. (Mianyang, China). 1,3-propanediol (PDO, 98%), poly(ethylene glycol) (PEG, M_n_, PEG: 2000 g/mol), and tetra butyl titanate (TBT, 99%, used as a catalyst) were purchased from Aladdin Reagent Co., Ltd. (Shanghai, China). All the materials were used without further purification.

### 2.2. Synthesis of PPFEG Copolymers and Corresponding PPF Homopolymer

Poly(propylene 2,5-furan dicarboxylate)-b-poly(ethylene glycol) (*PPFEG*) bio–based multiblock copolymers were synthesized via conventional two–step polycondensation. A typical procedure can be divided into two stages as follows. In the first stage, a mixture of DMFD and PDO with the first portion of TBT was added into a 250 mL three–necked round–bottom flask equipped with a distillation column and then purged with high purity nitrogen 3 times. The mixture was heated to 170 °C for 1 h and then heated to 180 °C for 5 h with continuous stirring. In the second stage, different dosages of PEG and the second portion of TBT were added to the reaction system. To reach the endpoint of the transesterification reaction, the mixture was heated slowly to 240 °C and held for 3 h under vacuum. After cooling to room temperature, the products were purified by dissolution in a mixed solvent of phenol and 1,1,2,2-tetrachloroethane (1/1, *w*/*w*) and then precipitated into excessive ethanol. The final products were dried in a vacuum oven at 70 °C for 1 day. The copolymer samples are denoted as PPFEG5, PPFEG10, and PPFEG20. For comparison, the PPF homopolymer was synthesized under similar reaction conditions as described above. Table 1 presents the molar amounts of each monomer used, as well as the weight–average molecular weight (M_w_) and dispersibility (M_w_/M_n_) of the polymers.

### 2.3. Thermal Characterization

Differential scanning calorimetry (DSC) measurements were carried out on a Discovery DSC series DSC25 (TA Instrument Corporation, New Castle, DE, USA) instrument calibrated using standards. The temperature was increased from 30 °C to 210 °C at 10 °C/min, and a constant temperature was maintained for 3 min to eliminate the thermal history. Then, the temperature was reduced to 0 °C at a cooling rate of 10 °C/min and increased to 210 °C at 10 °C/min after being maintained at 0 °C for 3 min [21].

The isothermal crystallization rate in the temperature range 90 ≤ *T*_c_ ≤ 130 °C was studied after rapid cooling (real cooling rate: 80 K/min) from 210 °C to *T*_c_ by analysing the heat evolved during crystallization as a function of time (t). Isothermal crystallization experiments were performed at temperatures between 90 and 120 °C after cooling the melt from 210 °C at 80 °C/min. The melting temperature of the formed crystals was evaluated upon subsequent heating at a rate of 10 °C/min. The samples were crystallized for sufficient time to obtain the leveling of the heat-flow-rate signal after the exothermal peak (60 min at 90 °C, 75 min at 100 °C, 90 min at 110 °C, and 180 min at 120 °C). The melting behavior was consecutive to isothermal crystallization in the temperature range 90 ≤ *T*_c_ ≤ 155 °C and was investigated by heating the purified PPF samples at 10 °C/min directly from *T*_c_ after complete crystallization to 210 °C. The Crystallinity was calculated according to the following equation:

$$X_{c}=\frac{{\Delta H}_{m}}{{\Delta H}_{m}^{0}}\times 100\%$$

where ΔH*_m_* is the enthalpy of melting, X*_c_* is the crystallinity, and ${\Delta H}_{m}^{0}$ is the theoretical melting enthalpy of 100% crystalline PPF.

Nonisothermal crystallization: 10 °C/min to 210 °C, constant temperature for 3 min, then 10 °C/min, 5 °C/min, 2 °C/min, 1 °C/min down to 0 °C, constant temperature for 3 min, then 10 °C/min to 210 °C again.

### 2.4. Wideangle X-ray Scattering Characterization

Wide–angle X-ray diffraction (WAXD) measurements were carried out on a PANalytical X’pert Pro diffractometer (PANalytical, Almelo, the Netherlands) with Cu Kα radiation (λ = 0.1546 nm) at room temperature, with a scanning angle 2θ range from 5° to 65°and a scanning rate of 20°/min, under a selected voltage and current of 40 Kv and 200 Ma, respectively. A PANalytical X’ Pert Pro diffractometer (PANalytical, Almelo, the Netherlands) equipped with a copper radiation source (λ = 1.5418 Å) and a fast solid-state X’Celerator detector were used. Data were acquired in the reflection mode using an Anton Paar TTK-450 (Anton Paar Trading Co., LTD., Shanghai, China) sample thermal stage.

The crystalline structures of the PPF samples after melting crystallization at *T*_c_ = 90 and 140 °C were analysed under non–isothermal conditions. First, the samples were heated at 30 °C/min from room temperature to their respective crystallization temperatures (*T*_c_ = 90 and 140 °C, respectively). Immediately afterward, the samples were heated from 90 or 140 °C to 170 °C at two °C/min, and WAXS scans were performed every 10 °C during the heating scans. The samples were heated from room temperature to melt temperature under isothermal conditions and then transferred to a hot table where the crystallization temperature was reached for crystallization, and when their crystallization was complete, they were brought down to room temperature and then subjected to XRD testing.

### 2.5. Morphological Characterization

The isothermal crystallization morphology of all the samples was observed using polarized optical microscopy (POM) (Olympus BX51-P) coupled with a computer–controlled CCD camera (Tota, Japan). A dual hot stage (MP41, Guangzhou Mingmei Electronics Co., Ltd., Guangzhou, China) was employed to control the isothermal crystallization temperatures of all samples. All the samples for observation were prepared by melt pressing. The coverslip was placed on a hot table, and when the temperature of the hot table reached the melting point, the sample was placed on the coverslip when it was completely melted. Another coverslip was placed on top of the sample, and the two coverslips were pressed flat into shape together with the sample.

## 3. Results and Discussion

### 3.1. Composition of PPF and PPFEG Copolymers

**Figure 1:**
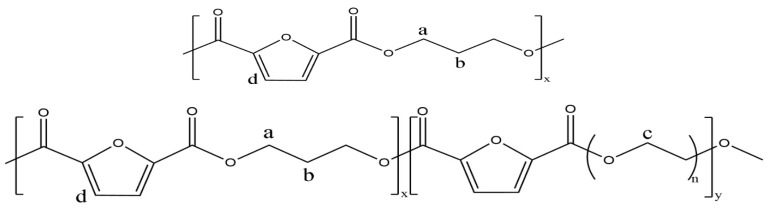
Chemical structures of PPF and PPFEG copolyesters.

**Figure 2:**
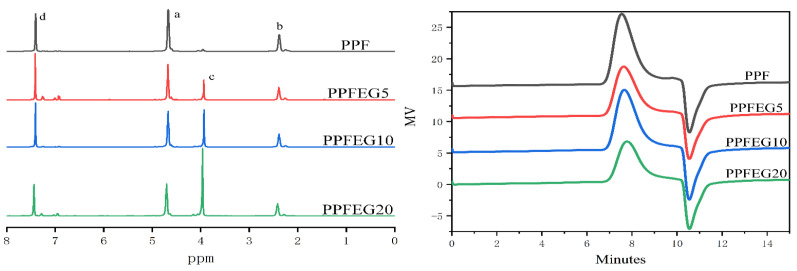
^1^H NMR and molecular weight curves of PPF and PPFEG copolyesters.

Figure 1 shows Chemical structures of PPF and PPFEG copolyesters. Figure 2 shows the chemical structures of PPF and PPFEG with the 1H-NMR results. The letters in the structural formula correspond to the letters of the nuclear magnetic hydrogen spectrum. For PPF, the absorption peak at the chemical shift of 7.41 ppm is the H atom on the furan ring (d); the absorption peaks at the chemical shifts of 2.38 ppm and 4.67 ppm are attributed to the methylidene hydrogen atom in the middle (b) and the hydrogen atoms at the ends (a) of CH_2_CH_2_CH_2_ in the PPF segment, respectively. The absorption peak at a chemical shift of 3.94 ppm was attributed to the methylene hydrogen atom in the PEG chain segment(c).

The ^1^H NMR hydrogen spectra of PPFEG copolymers also retained the characteristic peaks of PPF and PEG, and the characteristic peak areas of the PEG chain segments increased exponentially with increasing PEG content. The above structures indicate that PPFEG copolymers were successfully synthesized. As shown in Table 1, PPF and PPF copolymers were successfully synthesized with relatively large molecular weights, and their molecular weight distribution did not vary significantly.

### 3.2. Thermal Behavior of PPFEG Copolymers

#### 3.2.1. DSC Characterization of PPF and PPFEG Copolymers

**Figure 3:**
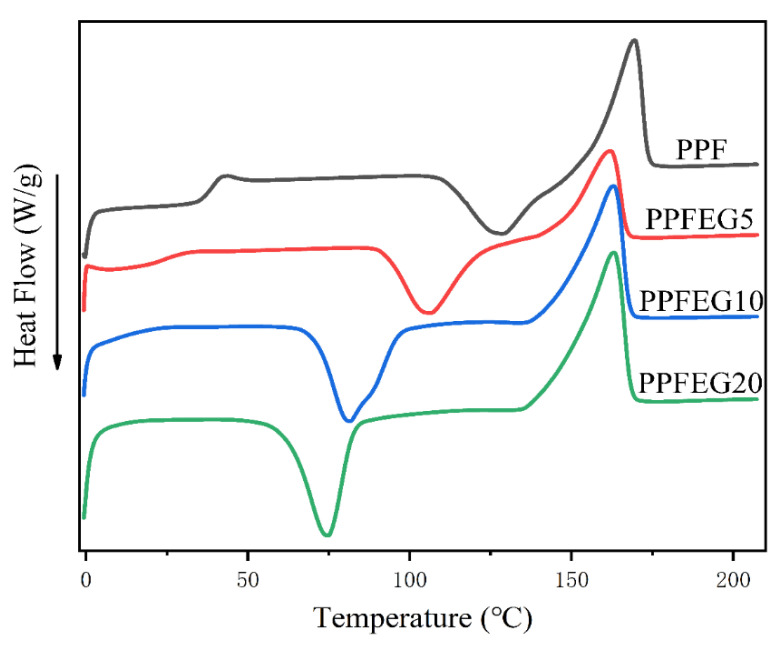
DSC curves of PPF and PPFEG copolymers.

Figure 3 shows the DSC curves of PPF and PPFEG copolymers heated at 10 °C/min. Pure PPF has an obvious T_g_ with a melting T_m_ of 169 °C and a crystalline peak temperature of 127 °C, showing a weak cold crystalline behavior. 

As can be seen from Table 2, the melting points of PPFEG5, PPFEG10, and PPFEG20 are 163.9 °C, 163.2 °C, and 163.1 °C, respectively, which are lower than the melting point of pure PPF. On one hand, PPFEG exhibits a significantly lower melting point as the proportion of polyethylene glycol chain segment (PEG) copolymerization units increases. This is due to the introduction of a flexible PEG structure through copolymerization, which results in a lower molecular weight of the hard segment PPF, leading to a lower melting point. However, the cold crystallization peaks of PPF and PPFEG decreased, and due to the weak crystallization ability of PPF, the overall motility of the molecular chain was enhanced, disrupting the regularity of the molecular chain. However, as the proportion of poly(ethylene glycol) chain segment (PEG) copolymerization units increased, ΔH*_m_* and X*_c_* of PPF increased. This was due to the increase in the degree of stability of the crystals due to the increase in the regularity of the crystals caused by block copolymerization, which leads to an increase in ΔH*_m_* and X*_c_*.

#### 3.2.2. Isothermal Crystallization of PPFEG Copolymers

**Figure 4:**
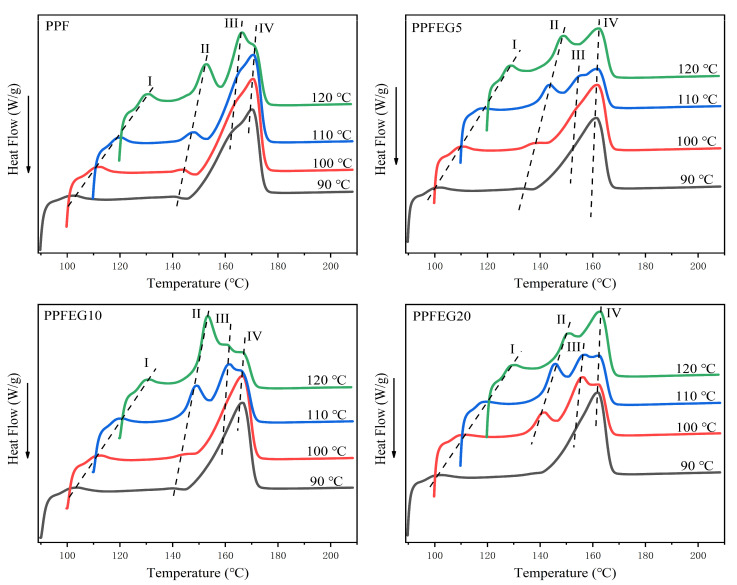
Melting curves of different *T*_c_ isothermal crystallization of PPF and PPFEG copolymers.

Figure 4 shows the melt curves of the PPF and PPFEG copolymers crystallized isothermally at different specified *T*_cs_ at a cooling rate of 80 °C/min. As shown in Figure 4, the melting point of PPF is 169 °C, which is close to 170 °C. The triple melting peak of pure PPF started to appear at 140 °C during the heating process and presented a multistage melting behavior. This is a multistage heat absorption and exothermic process, and a large single–crystal absorption peak appears at approximately 168 °C. The crystal melting of the PPF copolyester was relatively complete, which confirmed that there was a certain degree of crystal imperfection in the PPF melting process.

In Figure 4, there is a melting peak at 160–170 °C, but with an increase in the crystallization temperature, it slowly changes into two melting peaks. This behavior may be due to the formation of two structurally similar crystal modifications, which can be named *α*’ and *α*. The form of *α* may be associated with a loose and disordered crystal structure and can grow at the lowest crystallization temperature (90 ≤ *T*_c_ ≤ 100 °C), whereas *α* modification can be described as a perfect structure that can develop at higher temperatures (120 ≤ *T*_c_ ≤ 130 °C). With increasing crystallization temperature, the defect *α*’-phase is transformed into a more perfect *α*-form crystal. This may be because PPF contains more CH_2_ units in its chain, contributing to the conformational rearrangement required for crystal transformation.

In Figure 4, Ⅰ is the annealing peak, and I, III, and Ⅳ are the melting peaks from lower to higher temperatures. The reasons for these multiple melting behaviors are complex. Ⅱ and III are mainly related to the melting behavior of *β*-crystals, where Ⅱ increases with increasing *T*_c_ and Ⅲ barely shifts with increasing *T*_c_. This can be explained by the melting–recrystallization –remelting theory because imperfect *β*-crystals formed at higher *T*_c_ are more stable and have a shift in melting point towards higher temperatures, whereas, for the melting peak, Ⅲ of the perfect *β*-crystals formed by recrystallization does not change with increasing *T*_c_, a phenomenon similar to that reported by Chen [22]. Due to the restricted interval of melt recrystallization of PPF and PPFEG copolymer cool due to increasing *T*_c_, the intensity of all polyester Ⅲ decreases continuously and eventually overlaps with the progressively higher temperature shifted Ⅱ or higher temperature Ⅳ figure and disappears.

**Figure 5:**
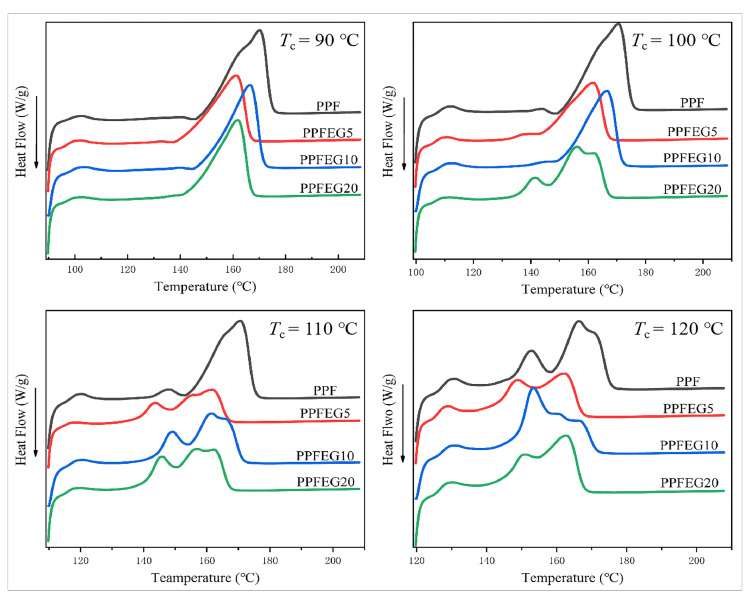
Isothermal melting curve of PPF and PPFEG copolymers at the same *T*_c._

Figure 5 shows the isothermal melting curves of the PPF and PPFEG copolymer at the same *T*_c_ at a temperature reduction rate of 80 °C/min. As seen from d in Figure 5, during the isothermal melt crystallization process, it can be first observed that the melt crystallization behavior of PPF above 120 °C is a triple melt absorption and exothermic process, but the triple melt peak of the copolymer appears earlier after adding different doses of PEG, a phenomenon similar to that reported by Wang [23]. An increase in the dose of PEG resulted in the early appearance of multiple melt crystallization behaviors of the polyester at 100 °C. All the crystallization peaks in the PPFEG polyester gradually became obvious with an increase in the dose of PEG added, and the lower the *T*_c,_ the more pronounced the main crystallization heat absorption peak was enhanced, and its crystallization ability became weaker. The lower the *T*_c_ and the higher the dose of PEG, the more pronounced the multistage melt crystallization behavior of the copolymer and even the formation of triple melt crystallization behavior, and the lower the main crystallization heat absorption peak of the copolymer, which is indicative of the increased perfection of the PPF crystals due to the addition of PEG.

#### 3.2.3. Non–Isothermal Crystallization of PPFEG Copolymers

The non–isothermal crystallization process more closely resembles the crystallization process of polymers in actual processing (extrusion and injection molding). The melting behavior of PPF after crystallization exhibits a triple melt peak, labeled as low, medium, and high–temperature melt.

**Figure 6:**
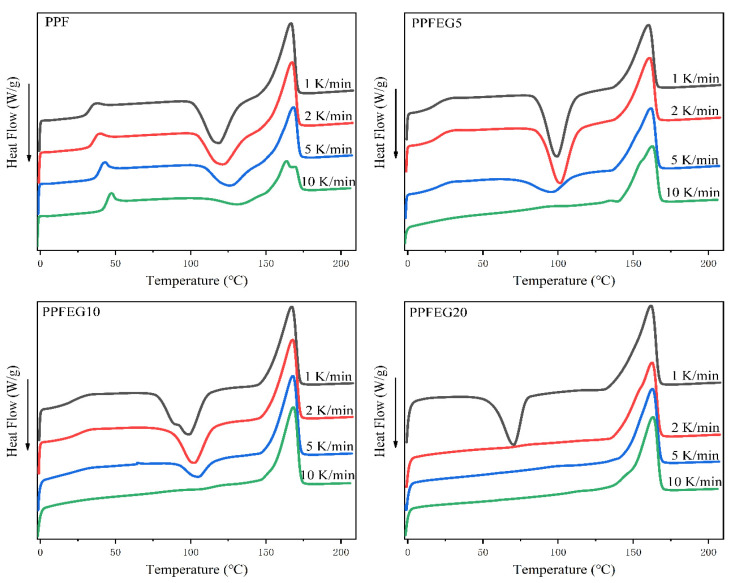
Secondary melting curves of PPF and PPFEG copolymers at different cooling rates peaks, depending on the corresponding peak temperature. Secondary melting of PPF and PPFEG copolymers after cooling to 0 °C at different cooling rate curves.

As you can see from Figure 6, the results show that PPF exhibits a double melt peak at a cooling rate of 1 °C/min while the low–temperature melt peak corresponding to the primary crystals is small and difficult to observe. In addition, the recrystallization peak temperature of the mid-temperature melt peak decreased and became weakened as the cooling rate increased. This is because the high cooling rate inhibits the recrystallization of the PPF segment in the copolymer. At the same cooling rate, the high–temperature melt peak of the copolymer decreases with increasing PEG content, and the medium–temperature melt peak becomes more obvious. As the PEG content increased, the mid–temperature melt peak gradually weakened and eventually merged with the high–temperature melt peak. This is mainly because the PEG segment in the copolymer facilitates the recrystallization of the PPF segment under non–isothermal crystallization conditions.

### 3.3. Crystalline Morphology of PPFEG Copolymers

**Figure 7:**
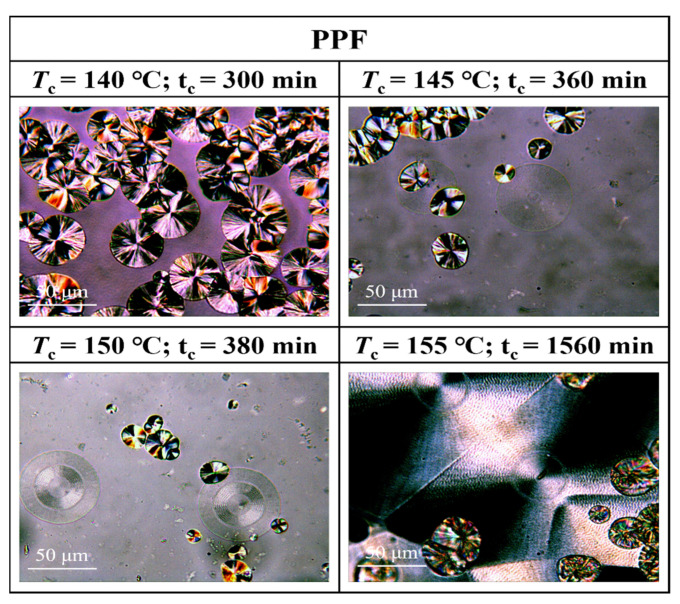
POM plots of PPF at different *T*_cs_ and crystallization times.

**Figure 8:**
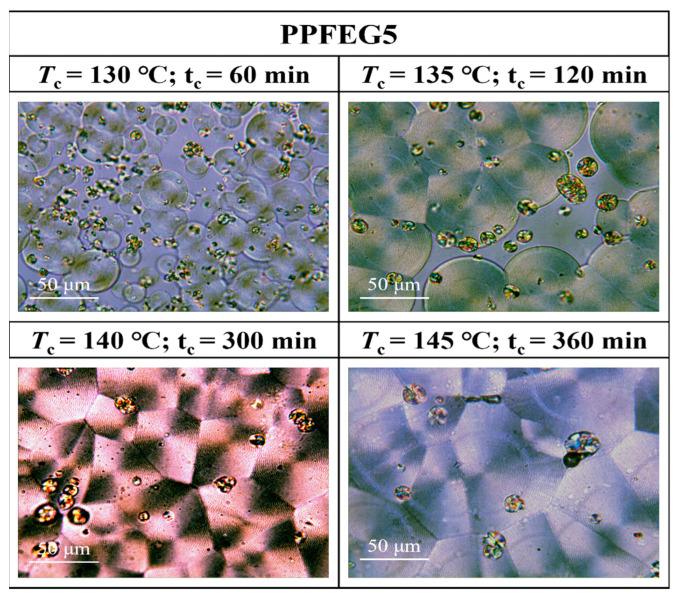
POM plots of PPFEG5 at different *T*_cs_ and crystallization times.

**Figure 9:**
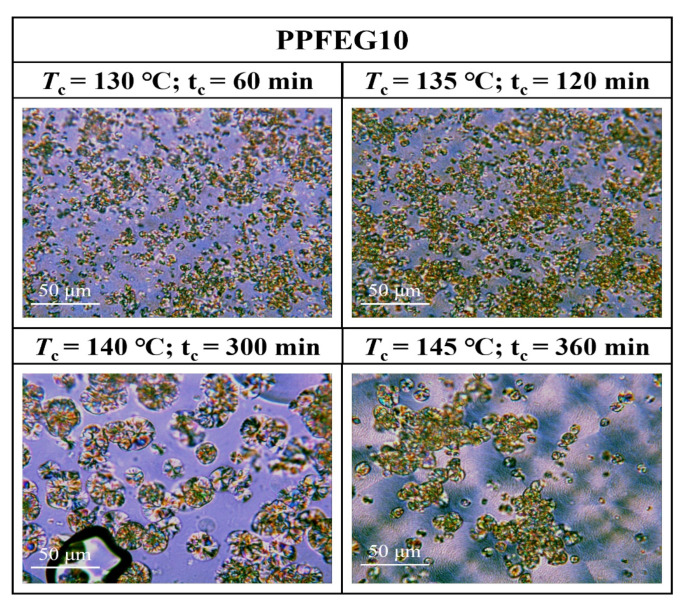
POM plots of PPFEG10 at different *T*_cs_ and crystallization times.

**Figure 10:**
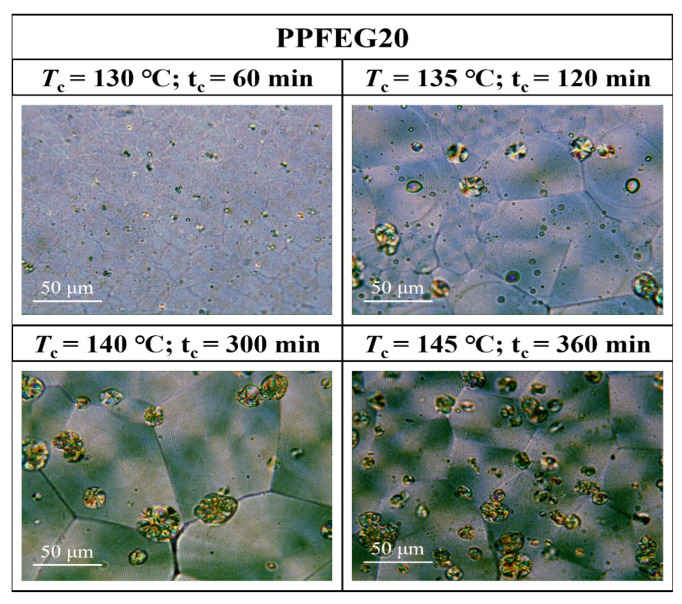
POM plots of PPFEG20 at different *T*_cs_ and crystallization times.

Figure 7, Figure 8, Figure 9 and Figure 10 show the spherical crystal morphologies of the PPF and PPFEG at different *T*_cs_ values and crystallization times. The results showed that the spherical crystal size of PPF and PPFEG increased, and the nucleation density decreased as the *T*_c_ increased. Because the *T*_m_ of PPFEG decreased significantly with increasing the proportion of PEG chain segments, which led to a decrease in the degree of supercooling, the nucleation density decreased significantly with increasing PEG chain segments, and the size of spherical crystals became larger at the same *T*_c_.

On the one hand, the proportion of *α* spherical crystals increased with increasing temperature, and the crystalline body of the PPF was completely transformed into *α* spherical crystals at 140 °C, which is similar to the results of the DSC isothermal crystallization kinetics analysis. However, the main body of crystallization gradually changes to *α*-spherical crystals with increasing PEG content. This was due to the increased molecular chain motility with the introduction of flexible chain segments, which led to an increase in the density of spontaneously nucleated *α*-spherical crystals. Because the growth rate of *α*-spherical crystals was significantly higher than that of *β*-spherical crystals, the growth of *β*-spherical crystals was inhibited. In PPFEG20, α-spherical crystals become the main body, and their overall crystallization rate is significantly increased.

It is noteworthy that the growth rate of *α* crystals of PPF and PPFEG is much higher than that of *β* crystals, but the nucleation density of *β* crystals is conversely higher than that of *α* crystals for PPF. With increasing *T*_c_, the nucleation density of both decreases to different degrees, but *β* crystals are inhibited to a more pronounced extent. At *T*_c_ = 140 °C, the main body of PPF has been transformed into *α* crystals, a finding consistent with the conclusion of Righettil [17]; therefore, the polycrystalline form of PPF can be controlled by adjusting *T*_c_.

**Figure 11:**
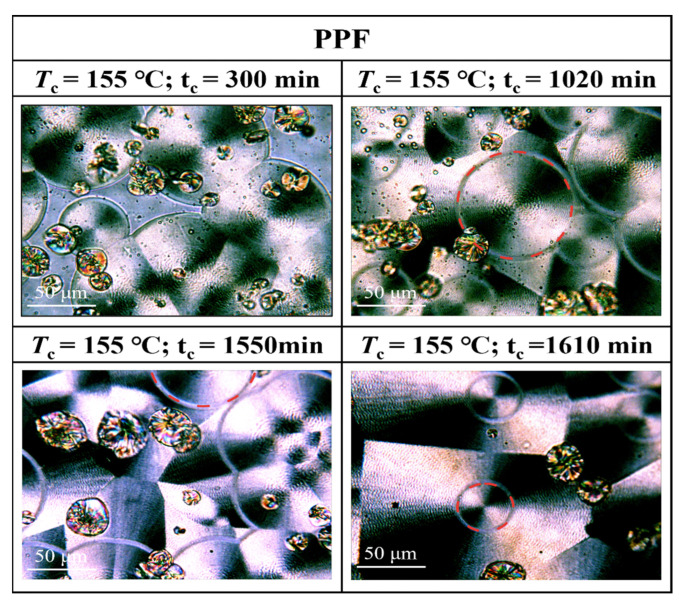
POM plots of PPF at 155 °C and different crystallization times.

**Figure 12:**
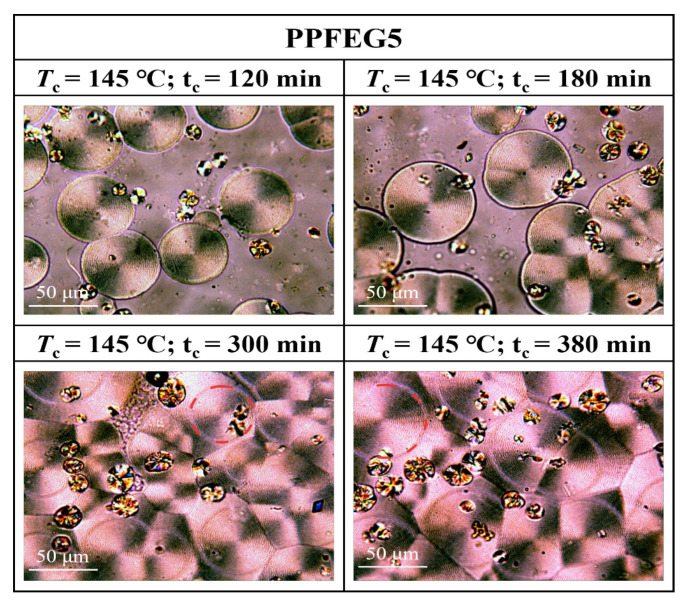
POM plots of PPFEG5 at 145 °C and different crystallization times.

**Figure 13:**
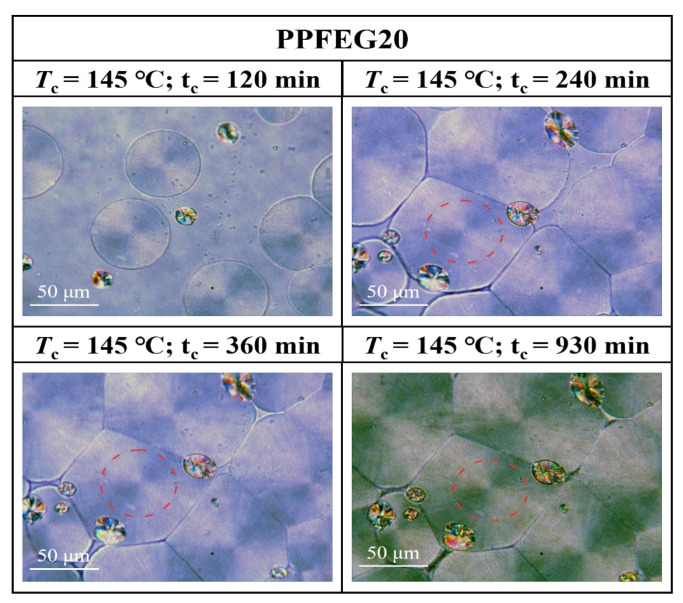
POM plots of PPFEG20 at 145 °C and different crystallization times.

Morphological transition from globular-free crystals to annular spherical crystals is a common phenomenon in the crystallization of polymers. Represented by the red circles in Figure 11, Figure 12 and Figure 13 are special cyclic ring-banded spherical crystals. Ring–shaped spherulites are shown in Figure 11, Figure 12 and Figure 13. From Figure 10, it can be seen that the PPF at a crystallization temperature of 155 °C slowly increases with increasing crystallization time, with the grain boundaries extending outwards. At a crystallization temperature of 155 °C, PPF starts with no special ring–like ring–banded spherical crystals at the beginning, but as the crystallization time increases, PPF starts to show special ring-like ring-banded spherical crystals. Because of the subcooling degree, we analysed the change in spherical.

Crystals of PPFEG5 and PPFEG20 were compared to the change in spherical crystals of PPF at a crystallization temperature of 145 °C. It can be seen from Figure 12 and Figure 13 that at a crystallization temperature of 145 °C, the spherical crystals of PPFEG5 and PPFEG20 will, like PPF, slowly increase in size as the crystallization time increases, but PPFEG5, like PPF, extends the grain boundaries of the crystals outwards as the crystallization time increases, whereas PPFEG20 shows no change in the position of the grain boundaries. As the PEG content increased, the addition of a small amount of PEG did not prevent the elongation of the grain boundaries, whereas the addition of an increased PEG content prevented the elongation of the grain boundaries. However, the addition of PEG reduced the crystallization time of PPF. The doping of PEG chain segments can greatly affect the crystallization ability of PPF chain segments, and PEG chain segments have a plasticizing effect on the PPF chain segments and enhance the mobility of PPF chain segments. As a result, the crystallization of PPF fragments is promoted so that the cyclic ring–banded spherical crystals appear earlier.

### 3.4. WAXD Characteristics of PPFEG Copolymers

#### 3.4.1. WAXD Patterns of PEG

**Figure 14:**
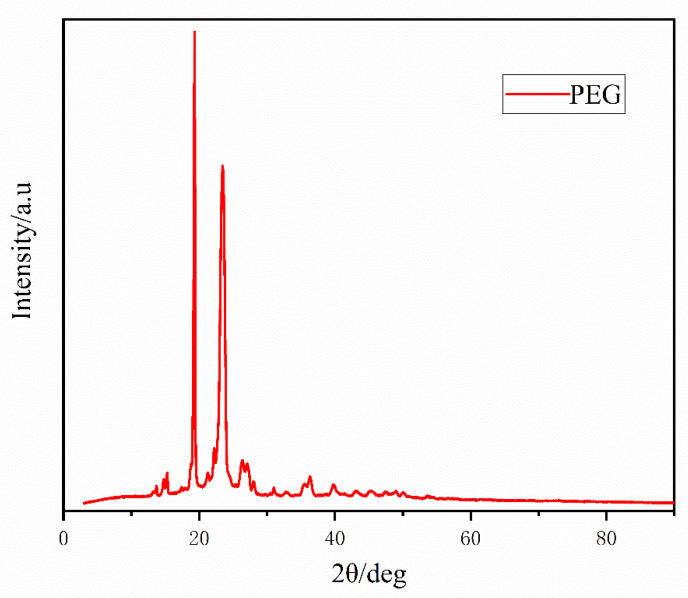
WAXD diagram of PEG.

From Figure 14, it can be seen that the XRD curves of pure PEG contain two crystalline diffraction peaks with higher intensities, with the 2θ angles located at 19.32 and 23.5°, respectively, and in addition, there are also crystalline diffraction peaks with lower intensities in the range of 2θ = 10~60° at 15.28°, 26.22°, 27.12°, 31.02°, 36.46, and 39.72° for PEG.

#### 3.4.2. WAXS Patterns upon Heating as a Function of the Isothermal Crystallization Temperature

**Figure 15:**
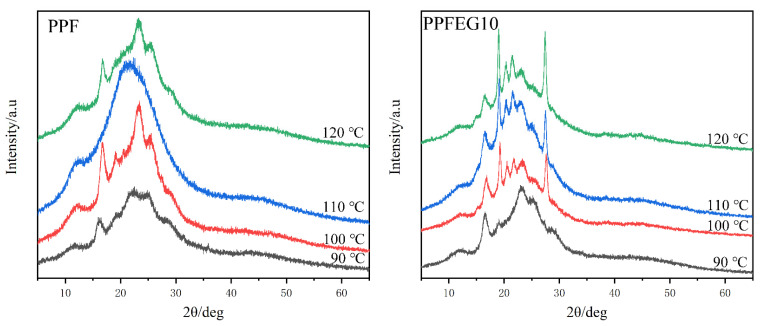
WAXD diagram of isothermal crystallization of PPF and PPFEG copolymers.

As you can see from Figure 15, the XRD patterns of the PPF samples crystallized at *T*_c_ = 90, 100, 110, and 120 °C have six major broad diffraction peaks at 2θ = 11.8, 15.9, 19.1, 22.3, 25.1, and 42.8° and an additional large peak at approximately 28.9° which overlaps with the reflections of the catalyst. The 16.4, 22.2, and 25.0° peaks are of comparable intensity, while the reflection at 19.1° is significantly smaller. A very broad reflection is observed at approximately 22°, which may be due to the overlap of the 22.2° and 23.0° peaks. The width and low intensity of all observed reflections prove that the crystalline structure formed by the PPF is essentially imperfect. The broadening of the reflections and reduction in peak intensity are generally caused by the structural disorder, which occurs when crystallizable units are packed in crystals in orientations and conformations that produce lattice distortion. New diffraction peaks appeared as the PEG content increased, as shown in Figure 14c,d. For the PPFEG copolymer, new reflections are observed at 14.9, 20.3, 21.5, and 27.6°. No new emissions appeared for PPFEG5, probably because there was no crystallization.

#### 3.4.3. WAXS Patterns upon Heating as a Function of the Non–Isothermal Crystallization Temperature

The crystal structures of the melt–crystallized PPF samples were analysed after WAXS heating at 2 °C/min to investigate the crystal structures at different crystallization temperatures and during warming.

**Figure 16:**
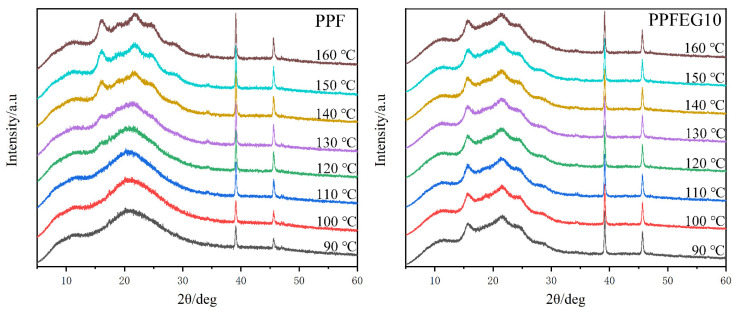
WAXD diagram of the non–isothermal crystallization of PPF and PPFEG copolymers.

As you can see from Figure 16, the XRD pattern of PPF at *T*_c_ = 90 °C had four main diffraction peaks located at 2θ = 11.8, 21.7, 39.1, and 45.7°. As the temperature slowly increased, new diffraction peaks appeared at 16.1, 18.9, 24.8, and 28.6°. The XRD pattern of PPF at *T*_c_ = 140 °C has eight major diffraction peaks at 2θ = 11.8, 16.1, 18.9, 21.7, 24.8, 28.6, 39.1, and 45.7°. No other new diffraction peaks appeared as the temperature was increased. This may be due to crystallization at low temperatures resulting in a single crystalline form (*β*-form), whereas at higher temperatures, two different crystalline forms grow and coexist in the purified PPF. The XRD pattern of PPFEG10 at *T*_c_ = 140 °C had seven major diffraction peaks located at 2θ = 11.8, 15.6, 21.7, 24.8, 28.6, 39.1, and 45.7°. However, as the temperature increased, no new diffraction peaks appeared, probably because the addition of PEG induced the crystallization of PPF, resulting in the growth and coexistence of two different crystal forms, even at lower temperatures.

## 4. Conclusions

(1) The structure of PEG consists of repeated glycol units embedded in PPF as a soft segment material, and the addition of PEG does not change the crystal structure of PPF. However, the addition of PEG affects the crystal structure of the copolymer to a certain extent and enhances its crystallization ability, and a PEG will inhibit the crystallization of the copolymer to a certain extent.
(2) The isothermal crystallization and melting behavior of the PPFEG polyester shows that when the *T*_c_ temperature continues to increase, the polyester crystal structure gradually becomes perfect. The PPF copolymer enhanced the crystallization ability of the molecular chains, and the crystallization rate was accelerated. The multiple melt crystallization behavior of the copolymers gradually becomes increasingly pronounced, as seen by their enhanced crystallization ability at this crystallization temperature compared to pure PPF.
(3) The addition of PEG decreased the crystallization temperature of PPF but increased the crystalline perfection of the polyester. The crystallization rate of the bio–based PPFEG polyesters tended to increase and then decrease with increasing amounts of flexible chain segment PEG and *T*_c_.
(4) From the crystalline morphology, the *T*_m_ of PPFEG decreased significantly with increasing proportion of PEG chain segments, which caused the crystalline body to change gradually to *α*-spherical crystals. With increasing crystallization time, ring–band spherical crystals appear slowly, and spherical crystals will also increase slowly.

## Figures and Tables

**Table 1:** Molecular weights of PPF and PPFFEG copolymers.

	M_n_	M_w_	M_p_	M_z_	M_z_/M_w_
PPF	18,394	47,865	46,141	85,151	1.778986
PPFEG5	14,731	41,383	37,767	76,948	1.859430
PPFEG10	12,570	37,588	35,644	69,488	1.848691
PPFEG20	12,176	31,785	27,065	59,545	1.873384

**Table 2:** Thermal characterization data obtained by DSC.

Polymer	T_m_ (°C)	△H*_m_* (J/g)	X*_c_* (%)
PPF	169.5	25.956	18.28
PPFEG5	163.9	30.180	21.25
PPFEG10	163.2	37.599	26.48
PPFEG20	163.1	45.128	31.78

## Data Availability

Data are contained within the article.
